# Biomechanical stability analysis of transpedicular screws combined with sublaminar hook-rod system using the finite element method

**DOI:** 10.3325/cmj.2021.62.328

**Published:** 2021-08

**Authors:** Krešimir Saša Đurić, Josip Rauker, Hrvoje Barić, Ivan Pašalić, Ivan Domazet, Petra Barl, Jurica Sorić, Marin Stančić

**Affiliations:** 1 Department of Neurosurgery, Clinical Hospital Center Zagreb, Zagreb, Croatia; 2 Medical 3D Design, Zagreb, Croatia; 3 Zagreb University School of Medicine, Zagreb, Croatia; 4 Faculty of Mechanical Engineering, Zagreb University, Zagreb, Croatia

## Abstract

**Aim:**

To develop and test a new posterior stabilization system by augmenting the posterior hook-rod system with screws and rods.

**Methods:**

A biomechanical analysis was performed using the finite element method. The anatomical structures were modeled based on computed tomography data. Instrumentation (hooks, rods, and screws) was modeled based on the data obtained by 3D scanning. The discretized model was verified by converging solutions and validated against data from a previously published experiment. A Th12-L1 spinal segment was modeled and modified by removing the body of the L1 vertebra (corpectomy) and the entire L1 vertebra (spondylectomy). The model was additionally modified by incorporating stabilization systems: i) posterior stabilization (transpedicular screws and rods); ii) combined posterior stabilization with sublaminar hooks; and iii) combined anterior (titanium cage) and posterior (sublaminar hooks) stabilization. The rotation angles in each group, and the strains on each part of the three stabilization constructs, were analyzed separately.

**Results:**

The combined anterior and posterior stabilization system was the stiffest, except in the case of lateral bending, where combined posterior stabilization was superior. Stress analysis showed that the posterior stabilization system was significantly unloaded when augmented with a hook-rod system. A significant strain concentration was calculated in the cranially placed hooks.

**Conclusion:**

Stiffness analysis showed comparable stiffness between the tested and proposed stabilization construct. Stress analysis showed luxation tendency of the cranially placed hooks, which would most likely lead to system failure.

Several factors are to be considered when deciding on the type of treatment of spine fractures: biomechanical stability, deformity, and neurological injury (1-3). The prevailing consensus among surgeons is to operate on unstable fractures, ie, fractures accompanied by neurological injury, with damage to all three vertebral columns, or if instability, deformity, or neurological deficit are imminent. The aims of surgical treatment are decompression of neural structures, biomechanical stability, and spinal deformity correction (4-8).

Biomechanical stability is achieved by using stabilization systems; based on the application site they can be anterior, lateral, and posterior. Stabilization systems enable anatomical restitution, fusion, and fast recovery (9). Anchoring options for posterior stabilization systems include sublaminar hooks, sublaminar wires, and transpedicular screws. Sublaminar hooks and wires are traditionally combined with Harrington distraction rods, and transpedicular screws with rods or plates (10-12). The invention of the transpedicular screw with its three-columnar anchoring increased the possibility of anchorage loading and thereby of shortening the system; this all led to the birth of the posterior segmental stabilization system (2,12). Therefore, the current standard in thoracolumbar spine stabilization includes transpedicular screws combined with plates or rods (13). Combined anterior and posterior stabilization remains the method of choice for achieving stability in the treatment of a spinal column that has lost its ability of withstanding axial loading, eg, complete burst fracture that may include translation and rotation (2,14). A limitation of the combined anterior and posterior stabilization technique is its invasiveness (15).

We aimed to test the hypothesis that the combined posterior stabilization system incorporating transpedicular screws, sublaminar hooks, and rods had comparable performance with regard to displacement and stability to the combined anterior and posterior stabilization in a virtual model of L1 corpectomy and spondylectomy.

## METHODS

A biomechanical analysis of three stabilization systems was performed on a L1 corpectomy and spondylectomy model by using the finite element method. The analysis was performed with the finite element software ABAQUS, version 6.13-1 (Abaqus, Inc -2004 – Abaqus, Inc., Providence, RI, USA).

**Figure 1:**
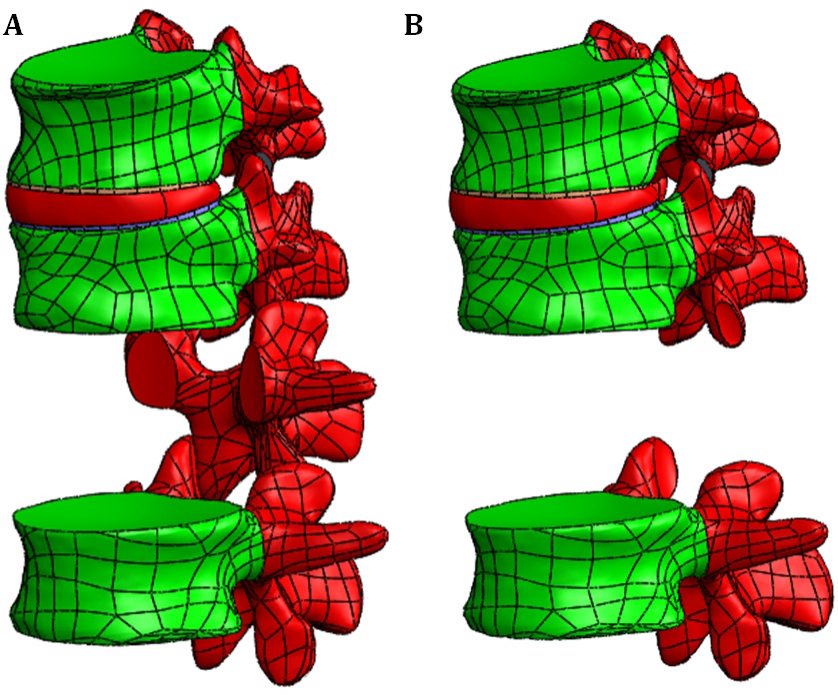
The corpectomy (**A**) and spondylectomy (**B**) model.

The L1 corpectomy model (Figure 1A) was used to simulate the relations in an anterior column biomechanical failure, and the spondylectomy model (Figure 1B) was used to simulate the relations in a three-columnar biomechanical failure. The models were tailored in a way that the stabilization system was maximally loaded, thus eliminating possible impact of the fractured fragments on load bearing.

**Figure 2:**
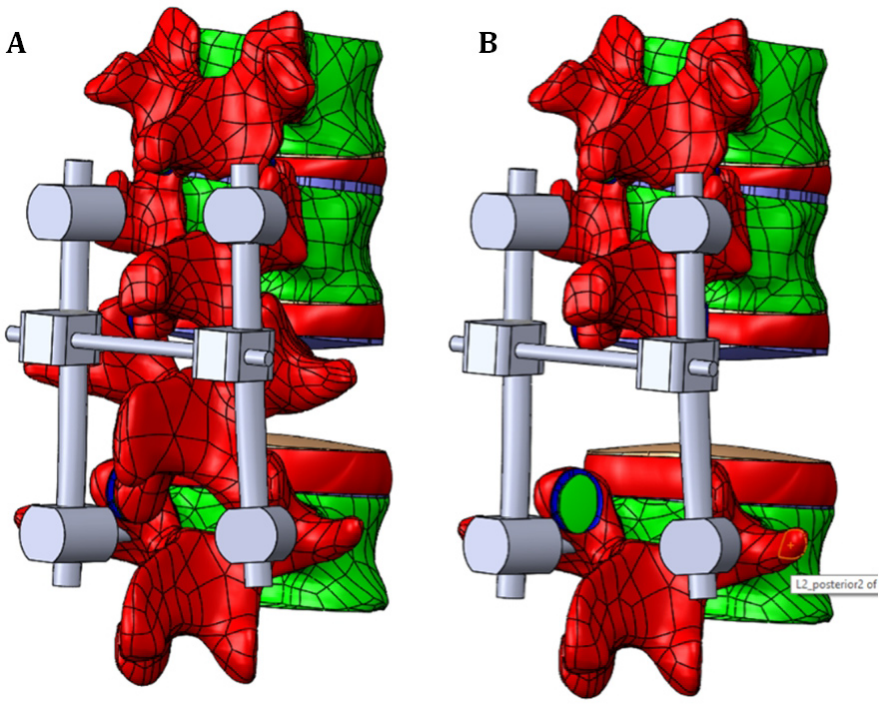
Posterior (transpedicular) stabilization on a corpectomy (**A**) and spondylectomy (**B**) model.

**Figure 3:**
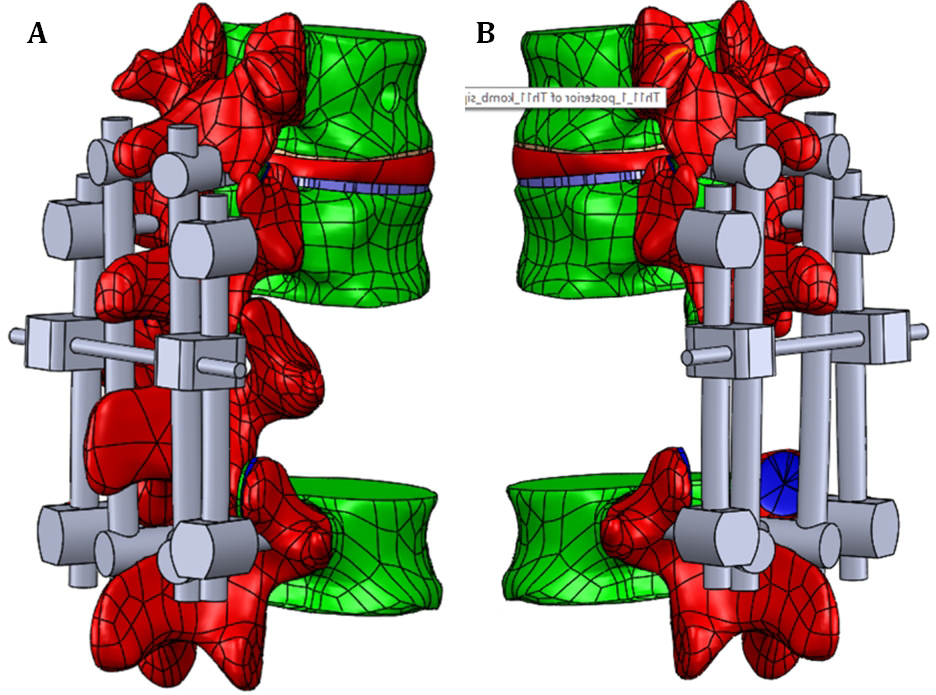
Combined posterior stabilization on a corpectomy (**A**) and spondylectomy (**B**) model.

**Figure 4:**
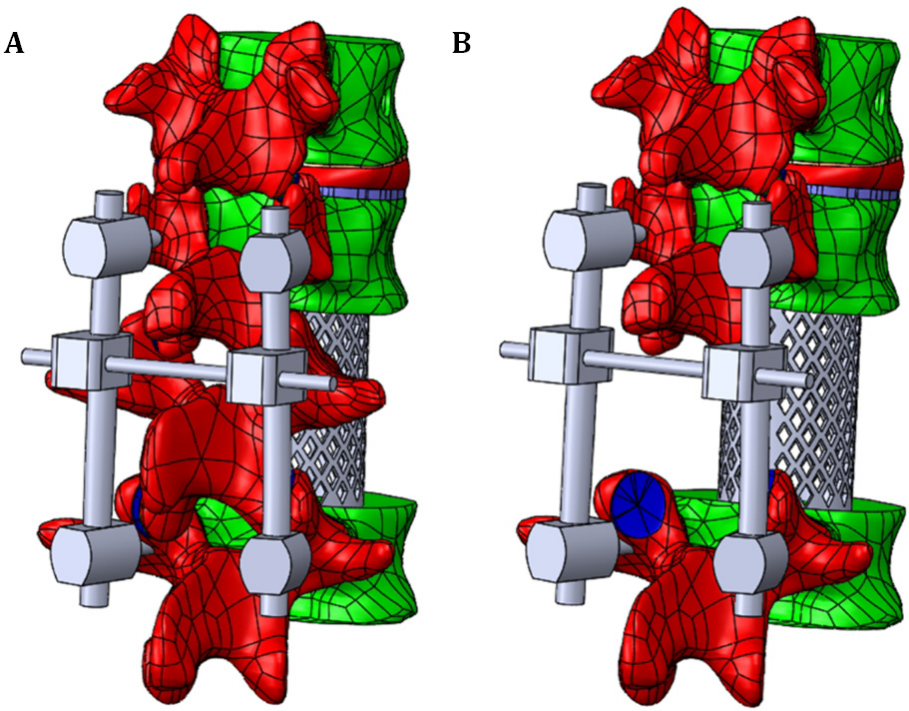
Combined posterior and anterior stabilization on a corpectomy (**A**) and spondylectomy (**B**) model.

Biomechanical properties of three stabilization systems were analyzed and compared on corpectomy and spondylectomy models: i) posterior stabilization system – transpedicular screws placed into the ThXII and LII vertebrae, connected by a longitudinal rod and a transverse link (Figure 2); ii) combined posterior stabilization system – posterior stabilization system combined with sublaminar hooks anchored into the cranial edge of L2 lamina and caudal edge of Th11 lamina and connected by a transverse link (Figure 3); iii) combined anterior and posterior stabilization system – posterior stabilization system combined with a titanium cylinder inserted between the vertebrae Th12 and L2 (Figure 4).

**Figure 5:**
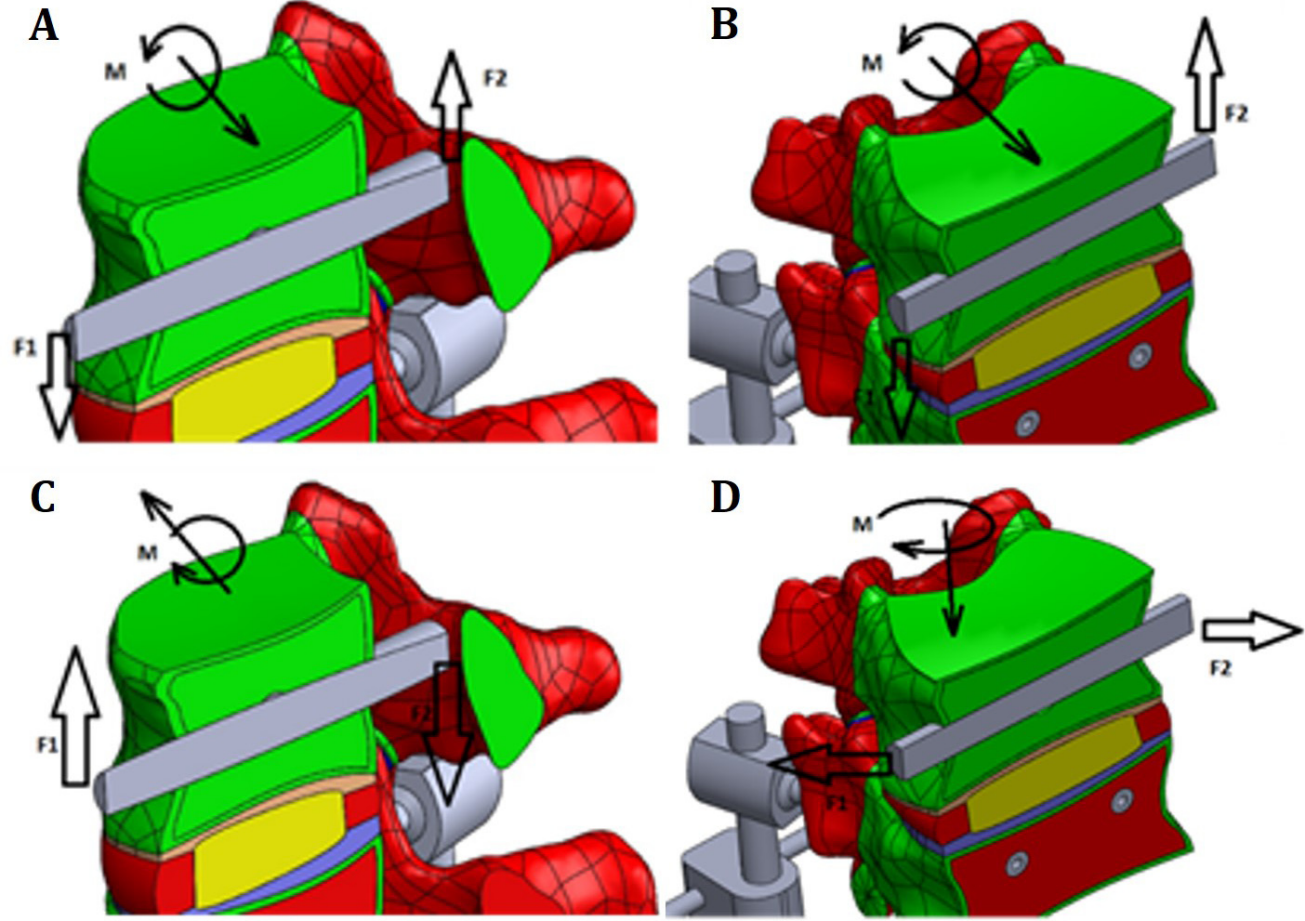
Force distribution on the steel rod inserted through the vertebral body Th11 for (**A**) flexion, (**B**) extension, (**C**) lateral bending, and (**D**) torsion: M – resultant momentum.

Node displacement was defined as a primary outcome. Displacement was calculated at four nodes at each loading cycle and each stabilization model. It was expressed in radians by calculating relative rotational displacements of specific points in the body of the Th12 vertebra during separate loadings. Next, stress distribution was analyzed during loadings on individual elements of the stabilization systems. The models were loaded by moments along three axes, leading to flexion, extension, and right-sided axial rotation (Figure 5). The bending moments were 1.356 Nm, 2.712 Nm, and 4.068 Nm, respectively. Von Mises stress was calculated and its distribution analyzed across separate loadings on individual elements of the three separate stabilization systems – this was defined as a secondary outcome.

The biological tissues were modeled based on data obtained by means of computerized tomography (CT) of a young multiple-traumatized patient with no injuries to the spine. Vertebrae were modeled as two separate units – vertebral body and posterior unit. Vertebral bodies were modeled as a composite of cortical and spongious bone, and posterior elements as an individual unit. Intervertebral discs were modeled so as to fit the intervertebral void produced by means of CT – the discs contain three separate elements (nucleus, annulus, cartilage end-plates). Inner tissue dimension were obtained from the literature (eg, bone thickness, end plate thickness, etc) (16). All the elements were assembled into a single Th11-L2 model. After the 3D model was created, it was discretized using hexahedral finite elements. Ligaments (anterior and posterior longitudinal, intertransverse, interspinous, supraspinous, capsular, and flavum) were modeled by rod finite elements with two nodes carrying only axial tension, yet not shear and compressive loads. Individual elements of the stabilization systems were modeled according to actual dimensions and physical properties. All model parts were defined as being linear elastic isotropic and homogeneous – elasticity modules and Poisson factors are available in the literature (17,18).

Before model validation, verification process of the model was assessed by determining the solution convergence for optimal finite elements density. Optimal node density was determined to be 282 885 hexahedral finite elements. Finally, the model was validated by comparing the calculated rotation angle at predefined specific points in the modeled functional spine unit Th12-L1 with experimental data from the 1972 Markolf experiment (19).

## RESULTS

### Rotation angle

The analysis of the rotation angle at four measuring points in each of the stabilization systems of the corpectomy and spondylectomy model showed variability. The variability was primarily due to differences in the positions of each point with regard to the center of rotation. Therefore, we considered it sufficient to present the results from a single point. We chose the point number 2, which corresponds to node number 2695, located at the ventral surface of the Th12 vertebra.

Displacement analysis at point 2 of the posterior stabilization system on the corpectomy and spondylectomy model did not show significant differences under three different loads. The difference during extension was 2.1%, during flexion 1.9%, during lateral bending 0.8%, and during torsion 5.1% (Table 1). Displacement analysis of the combined posterior stabilization of both models did not show significant differences. The difference during extension was 0.0%, during flexion 0.3%, during lateral bending 0.08%, and during torsion 0.0% (Table 1). Displacement analysis of the combined posterior and anterior stabilization also showed no significant difference. The difference during extension was 0.5%, during flexion 0.3%, during lateral bending 0.05%, and during torsion 0.04% (Table 1). Based on these results, and for clarity, displacement angles and strains occurring only in the case of spondylectomy were further analyzed.

**Table 1:** Rotation angles (in radians) of the three stabilization systems and a healthy spine for given moments: moment 1 – 1.356 Nm; moment 2 – 2.712 Nm; momentum 3 – 4.068 Nm, in order to achieve flexion, extension, lateral bending, and torsion, respectively*

	Extension		Flexion		Lateral bending		Torsion	
	S	C	S	C	S	C	S	C
Posterior stabilization								
moment								
1	0.072218182	0.070757576	0.071787879	0.070684848	0.006904848	0.00685515	0.043229091	0.043101818
2	0.144406061	0.141715152	0.143545455	0.141024242	0.013842424	0.01378	0.086612121	0.086369697
3	0.216587879	0.207248485	0.21530303	0.211418182	0.020801818	0.02171151	0.129442424	0.122090909
Combined posterior stabilization								
moment								
1	0.012461818	0.012462424	0.012007273	0.011977576	0.000784364	0.000783697	0.007576364	0.0.00767454
2	0.024343636	0.024881212	0.024010303	0.023961212	0.00156903	0.001566909	0.015355152	0.015352121
3	0.03716	0.037177576	0.036012727	0.035938788	0.002353455	0.002350242	0.02308303	0.023078182
Combined posterior and anterior stabilization								
moment								
1	0.002229091	0.002219394	0.002189697	0.00218303	0.002997212	0.002995758	0.00079903	0.000798788
2	0.004464848	0.004447879	0.004380606	0.004366667	0.005979273	0.005975758	0.001590848	0.001590909
3	0.006721212	0.006715152	0.006569697	0.006551515	0.008955758	0.008951515	0.002374303	0.002374545
Healthy spine								
moment								
1	0.039585455		0.036029091		0.054903636		0.029047273	
2	0.079187879		0.072072727		0.108284848		0.066509091	
3	0.118769697		0.10810303		0.160975758		0.097157576	

*Abbreviations: S – spondylectomy; C – corpectomy.

### Stiffness

Stiffness is defined as the relationship between the bending moment and displacement angle. Stiffness is proportional to the slope of the bending moment/displacement curve, which is equal to the tangent value of the angle between the curve and the horizontal axis.

**Figure 6:**
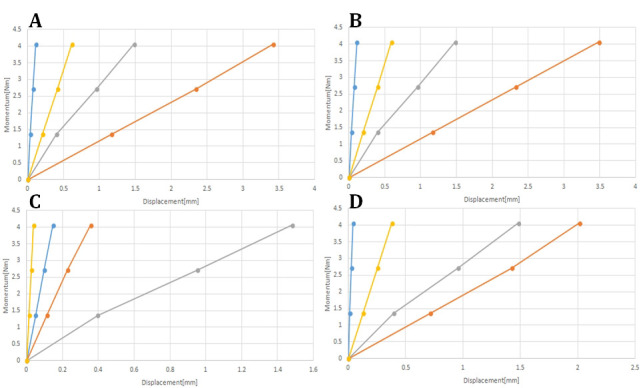
Moment vs rotation angle of the stabilization constructs (blue – combined anterior and posterior; orange – posterior; yellow – combined posterior) and the healthy spine (gray) on the spondylectomy model during (**A**) flexion, (**B**) extension, (**C**) lateral bending, and (**D**) torsion.

**Figure 7:**
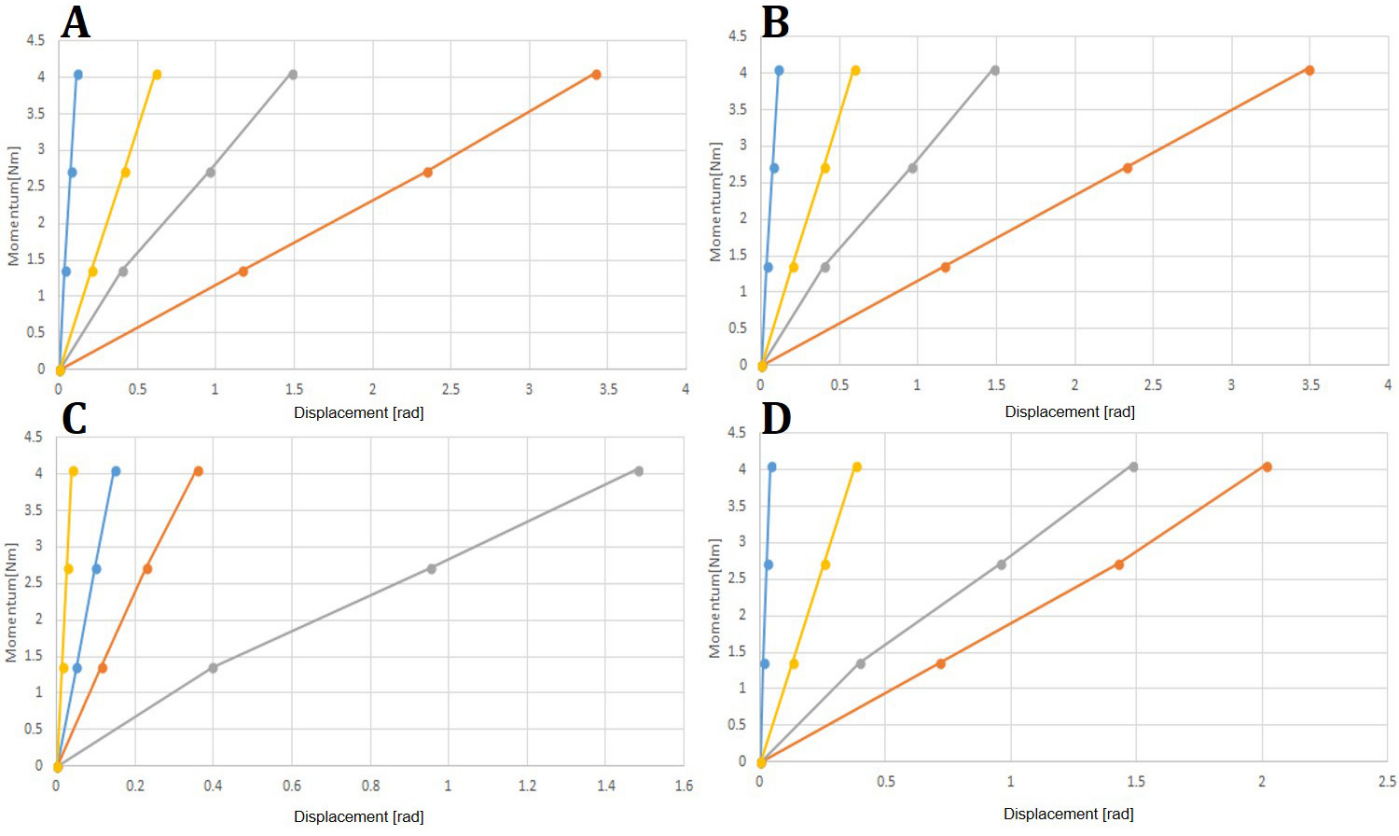
Moment vs rotation angle of the stabilization constructs (blue – combined anterior and posterior; orange – posterior; yellow – combined posterior) and the healthy spine (gray) on the corpectomy model during (**A**) flexion, (**B**) extension, (**C**) lateral bending, and (**D**) torsion.

Tangent values of the three stabilization systems are shown in Table 2. Spondylectomy and corpectomy did not affect the stiffness of the stabilization system (Figure 6, Figure 7). Different stabilization systems were ranked as follows (higher to lower stiffness): combined anterior and posterior stabilization, combined posterior stabilization, healthy spine, and posterior stabilization. The ranks were maintained during extension, flexion, and torsion. In the case of lateral bending, combined posterior stabilization was the stiffest construct.

**Table 2:** Tangent values of shift angles for the three stabilization systems (for both spondylectomy and corpectomy) and healthy spine

	Combined anterior and posterior	Posterior	Combined posterior	Healthy spine
**Extension**				
spondylectomy	608.32	18.78	108.81	34.26
corpectomy	610.98	19.16	108.81	
Flexion				
spondylectomy	619.26	18.89	112.93	37.64
corpectomy	621.15	19.18	113.21	
Lateral bending				
spondylectomy	452.42	196.38	1728.79	24.70
corpectomy	452.64	197.81	1730.26	
Torsion				
spondylectomy	1697.06	31.37	176.65	46.68
corpectomy	1697.57	31.46	176.69	

### Von Mises stress

Stress analysis in the screws of the three stabilization systems clearly showed lower stress on the screws of the combined anterior and posterior stabilization (Table 3). Stress values were comparable during extension, flexion, and lateral bending and amounted to approximately 20 MPa, and during torsion amounted to approximately 10 MPa. The highest stress values were calculated in the screws of the posterior stabilization, and amounted to approximately 300 MPa (flexion and extension), 250 MPa (torsion), and 90 MPa (lateral bending), with a significantly higher stress on the upper screws (upper 95 and 97 MPa, lower 64 and 67 MPa). In the screws of the combined posterior stabilization, the upper screws had higher stress values during flexion and extension (approximately 130 and 19 MPa). The results indicate that inserting rods and hooks unloads the upper screws of the combined posterior system, while the stress in the lower screws remains comparable to the values calculated in lower screws on the combined posterior and anterior stabilization.

**Table 3:** Maximum equivalent von Mises strains for individual construct elements of the combined anterior and posterior, posterior, and combined posterior stabilization (spondylectomy)*

	Extension				Flexion			Lateral bending			Torsion		
	360°	Post		Com	360°	Post	Com	360°	Post	Com	360°	Post	Com
Screws													
UR	22.37		354.27	147.58	25.99	355.45	145.33	24.6	95.74	99.11	8.02	243.91	201.49
UL	21.91		298.04	128.87	24.24	297.02	126.78	22.13	97.55	51.18	11.17	249.51	79.61
LR	17.47		287.3	18.52	17.41	287.07	18.75	20.4	64.8	22.66	12.54	246.69	70.15
LL	17.65		347.18	20.31	17.25	344.26	22.21	20.4	67.28	21.71	11.73	253.67	42.75
Longitudinal rods													
L1	15.33	127.3		42.31	15.66	127.54	41.8	17.6	71.89	12	6.27	83.5	14.43
L2	-	-		21.72	-	-	21.87	-	-	18.88	-	-	14.3
R1	13.91	121.75		51.5	14.19	121.96	50.98	19.4	61.1	12.11	7.68	77.53	16.35
R2				21.71	-	-	21.99	-	-	18.89	-	-	14.01
Transverse rod													
	6.92	17.45		8.29	9.51	18.75	8.63	17.78	56.63	15.09	34.68	201.94	36.31
Hooks													
UR	-	-		260.02	-	-	264.27	-	-	320.19	-	-	227.84
UL	-	-		232.49	-	-	235.83	-	-	442.17	-	-	254
LR	-	-		113.44	-	-	114.98	-	-	133.12	-	-	62.16
LL	-	-		132.7	-	-	133.65	-	-	135.94	-	-	56.24
Bone													
ThXII(s)	3.31	34.43		14.38	3.08	30.35	10.98	3.76	15.08	6.39	1.57	27.65	7.09
LII (s)	2.95	31.11		3.03	2.86	29.3	3.27	3.18	9.65	1.1	1.66	25.68	2.87
ThXI(h)	-	-		89.43	-	-	25.83	-	-	37.97	-	-	34.89
LII (h)	-	-		14.83	-	-	13.39	-	-	9.86	-	-	1.39

*Abbreviations: 360° – combined posterior and anterior stabilization; Post – posterior stabilization; Com – combined posterior stabilization; UR – upper right; UL – upper left; LR – lower right; LL – lower left; L1 – left rod of the posterior (transpedicular) construct; R1 – right rod of the posterior (transpedicular construct); L2 – left rod of the combined posterior (with hooks) construct; R2 right rod of the combined posterior (with hooks) construct; ThXII (s) – stress on the bone adjacent to the screw entry; LII (s) – stress on bone adjacent to bone entry; ThXI (h) – stress on bone adjacent to hook anchorage; LII (h) – stress on bone adjacent to hook anchorage.

We observed exceptionally high peak stress in the right cranial screw during lateral bending (99 MPa) and torsion (201 MPa). A similar pattern was observed for the left cranial hook (lateral bending 442 MPa and torsion 254 MPa). These findings might indicate weak spots of the system at which system element failure is most likely to occur.

The analysis of peak stress in the screws of individual stabilization systems showed a concentration of stress at the bone entry site, which corresponds to the first thread. The result was identical for all analyzed cases and is in accordance with classical mechanics postulates.

## DISCUSSION

Stress analysis of the three stabilization systems showed several major results. First, reconstructing the anterior column by inserting a titanium cylinder significantly unloaded the screws of the posterior segmental stabilization system. Second, by strengthening the posterior stabilization with rods and hooks, cranial screws were significantly unloaded during flexion and extension. At the same time, stress on caudal screws remained similar to the stress on caudal screws of the combined anterior and posterior stabilization system. In addition, augmenting the posterior system with hooks and rods brought about a significant asymmetrical unloading of the screws, more prominently the caudal screws. Third, stress on the screws of stabilization systems occurred at first threads, a finding consistent with classical mechanics postulates. Fourth, stress was concentrated on the upper hooks with asymmetry in calculated strains and the maximum value at the upper left hook during lateral bending and torsion. Fifth, stress on longitudinal rods was reduced by augmenting the stabilization systems with hooks and rods, thereby making the stress values comparable to the values for the combined anterior and posterior stabilization. Sixth, stress on bones correlated with the calculated stress in the screws and hooks of stabilization systems. Finally, the transverse rod added to system stiffness mostly during lateral bending and torsion. Unloading of the transverse rod was comparable between combined posterior and combined anterior and posterior stabilization.

Finite element method is based on physical discretization of a continuum. The continuum is discretized by a finite number of elements interconnected in points along the contours, called nodes of finite elements. Relations between the nodes are described by a function to determine the properties of the examined continuum – in this case, a biomechanical problem. The results of the analysis are therefore affected by: i) errors in the discretization of the continuum; ii) number and types of elements; iii) choice of function describing the properties of materials; iv) inadequately set extreme conditions and loadings; v) errors in describing the mathematical formulae; and vi) study design. The model was verified and validated to reduce the error margin.

Building the model of the thoracolumbar spine included model verification and validation. Verification was used to determine the optimal density of the final elements. Validation was performed as an *in silico* experiment re-creation and comparison of experimental relations of the bending moment and resulting displacement angle of characteristic points. Difference in the displacement angle was 5.39% for flexion, 43.98% for extension, 8.4% for lateral bending, and 82.8% for torsion. These results show that the created model is only partially accurate in representing the experimental movements in the thoracolumbar spine. The discrepancies can be explained by several factors, eg, a difference in modeling material properties and a difference in patient age (ie, spine) between the cadaveric model and the *in silico* model. These differences were considered while interpreting the results.

The range of motion analysis (ie, displacement angle and moments) showed negligible differences between different types of stabilization systems for both spondylectomy and corpectomy, which excludes the impact of mechanical properties of models of the thoracolumbar spine on stabilization system models. This result is expected and easily explained by the fact that titanium has an elasticity modulus of 114 000 N/mm^2^ and the cortical bone has an elasticity modulus of 12 000 N/mm^2^, which means that the load is transferred through the material with a higher elasticity modulus.

The results of the analysis are affected by two other approximations. The first is the definition of the contact point between end plates of the Th11 and L2 vertebrae and the titanium cylinder, and the contact point between hooks and laminae. The contact point between the end plates and the titanium cylinder was defined as being solid, which enabled simulating properties of the anterior stabilization system – it consists of a titanium cylinder and a plate applied on the sides of the vertebral bodies and held in place by bicortical screws. We believe that the approximation is close to the real solution; however, this was not tested. Further on, the contact of the hooks and the laminae was also defined as being solid, which is not the case. The contact has a certain degree of freedom, which contributes to lesser loads on the hooks.

Previous sections raise the questions of the study's internal and external validity, ie, the applicability of the results to everyday clinical practice. The results are of use when comparing the efficiency of the tested stabilization systems in a given setting, since the sources of errors are comparable between different systems. Absolute accuracy is accuracy in relation to the real situation.

Internal (relative) validity of the results is significant, since the models were compared between themselves, and they all have the same degree of error in individual computer models. In this way, conditions of a separate system were met, and the results are relative values, which can be accurately compared between them. The calculated results of the stabilization models, when compared to the thoracolumbar spine model, are descriptive in their nature, ie, they are relative and hardly absolute, which results in a limited external validity of the study.

As mentioned before, there are methods to assess the accuracy of the calculations, which is important for general applicability of the results of the numerical analysis. As well, it is important to compare the results with expected, ie, assumed events. For example, we assumed that the system of posterior stabilization would show the lowest stiffness and the highest loads on screws, that the system of combined anterior and posterior stabilization will be the stiffest system, and that loads on screws will be lowest – all these assumptions are in accordance with the results. The observed concentration of stress on the first thread of the screw is in accordance with the postulates of classical mechanics. Further, it is a known rule that the stabilization system should incorporate an additional level cranial to the fracture, as opposed to the caudal levels, which results in a significantly better stabilization systems stability (20). The results of the study can be considered applicable to general cases and clinical cases, due to a high correlation between the calculated and expected results. However, although the results cannot be considered applicable in an absolute sense, they are applicable in a sense of a trend and as an aid in understanding trends in stiffness and stress concentration. We are careful in the interpretation of our work, given the limitations discussed previously; however, we hold the results indicative as a strong model for future research.

As opposed to numerical analysis, laboratory experiments, in particular biomechanical experiments on the cadaveric spine, will yield accurate results for node displacement angles. Stress analysis results will surely have lower reliability due to technical possibilities and limitations arising from laboratory settings. Forces that would lead to stabilization systems failure would be easily measurable and analyzable. Results of such analyses have higher external validity and absolute accuracy; however the number of test samples would need to be substantial to account for different anatomical, physiological, and clinical variables. In real life, the surgeon applying stabilization systems considers variables that could lead to system failure and tailors the optimal stabilization system according to personal experience. Therefore, the single most important factor determining the optimal usage of stabilization systems is the surgeon's experience, arising from personal clinical practice, published experience, and experimental data.

Results of biomechanical analyses (*in silico* and *in vitro*) are not to be considered absolute values; rather they serve to clarify relations between forces in complex systems. Technological advances have enabled direct measurements of stress on individual elements of stabilization systems. This approach permits accurate results that give precise insight into relations between forces and could probably be used for early intervention in the cases of impending system failure (18,21,22).

In conclusion, stiffness analysis showed comparable stiffness between the tested and proposed stabilization system. Stress analysis showed luxation tendency of the cranially placed hooks, which would most likely lead to stabilization systems failure.
